# Neuroinflammatory and cognitive consequences of combined radiation and immunotherapy in a novel preclinical model

**DOI:** 10.18632/oncotarget.13551

**Published:** 2016-11-24

**Authors:** Gwendolyn J. McGinnis, David Friedman, Kristina H. Young, Eileen Ruth S. Torres, Charles R. Thomas, Michael J. Gough, Jacob Raber

**Affiliations:** ^1^ Howard Hughes Medical Institute, Oregon Health and Science University, Portland, OR; ^2^ Department of Behavioral Neuroscience, Oregon Health and Science University, Portland, OR; ^3^ Department of Radiation Medicine, Oregon Health and Science University, Portland, OR; ^4^ Earle A. Chiles Research Institute, Providence Cancer Center, Portland, OR; ^5^ Department of Neurology, Oregon Health and Science University, Portland, OR; ^6^ Division of Neuroscience, Oregon National Primate Research Center, Portland, OR

**Keywords:** neuroinflammation, cancer-related neurological dysfunction, microglia, immunotherapy, radiotherapy

## Abstract

**Background:**

Cancer patients often report behavioral and cognitive changes following cancer treatment. These effects can be seen in patients who have not yet received treatment or have received only peripheral (non-brain) irradiation. Novel treatments combining radiotherapy (RT) and immunotherapy (IT) demonstrate remarkable efficacy with respect to tumor outcomes by enhancing the proinflammatory environment in the tumor. However, a proinflammatory environment in the brain mediates cognitive impairments in other neurological disorders and may affect brain function in cancer patients receiving these novel treatments. Currently, gaps exist as to whether these treatments impact the brain in individuals with or without tumors and with regard to the underlying mechanisms.

**Results:**

Combined treatment with precision RT and checkpoint inhibitor IT achieved control of tumor growth. However, BALB/c mice receiving combined treatment demonstrated changes in measures of anxiety levels, regardless of tumor status. C57BL/6J mice with tumors demonstrated increased anxiety, except following combined treatment. Object recognition memory was impaired in C57BL/6J mice without tumors following combined treatment. All mice with tumors showed impaired object recognition, except those treated with RT alone. Mice with tumors demonstrated impaired amygdala-dependent cued fear memory, while maintaining hippocampus-dependent context fear memory. These behavioral alterations and cognitive impairments were accompanied by increased microglial activation in mice receiving immunotherapy alone or combined with RT. Finally, based on tumor status, there were significant changes in proinflammatory cytokines (IFN-γ, IL-6, IL-5, IL-2, IL-10) and a growth factor (FGF-basic).

**Materials and Methods:**

Here we test the hypothesis that IT combined with peripheral RT have detrimental behavioral and cognitive effects as a result of an enhanced proinflammatory environment in the brain. BALB/c mice with or without injected hind flank CT26 colorectal carcinoma or C57BL/6J mice with or without Lewis Lung carcinoma were used for all experiments. Checkpoint inhibitor IT, using an anti-CTLA-4 antibody, and precision CT-guided peripheral RT alone and combined were used to closely model clinical treatment. We assessed behavioral and cognitive performance and investigated the immune environment using immunohistochemistry and multiplex assays to analyze proinflammatory mediators.

**Conclusions:**

Although combined treatment achieved tumor growth control, it affected the brain and induced changes in measures of anxiety, cognitive impairments, and neuroinflammation.

## INTRODUCTION

Behavioral and cognitive impairments are a common and devastating complication of cancer and cancer treatment [1–3]. These impairments often include difficulty concentrating, memory impairments, fatigue, and increased anxiety [4]. Cancer-related behavioral changes and cognitive impairments often last well beyond treatment, with up to 35% of patients reporting effects lasting months or years [5]. Given improved cancer outcomes and long-term survivorship, these impairments are becoming increasingly important to study. Cancer-related neurological dysfunction, which may contribute to a suboptimal functional quality of life, has been widely reported in human patients, yet the underlying mechanisms remain elusive.

Historically, these impairments have been associated with cytotoxic chemotherapy (“chemobrain”). However, these changes have also been identified in patients who have not yet received treatment or received peripheral (non-brain) radiation alone, demonstrating that these changes are most likely multifactorial [6–10]. One such factor that has come under recent scrutiny is the role of the immune system [11–15].

Neuroinflammation has been identified as a mediator of behavioral alterations and cognitive impairments in many neurological conditions, including Alzheimer's Disease, Major Depression, and injury from cerebral ischemia [16–18]. The expression of key proinflammatory mediators such as TNFα, IL-1β, IFN-γ, and IL-6 have been associated with mood disorders and their administration has been shown to induce depressive symptoms [19]. In addition to enhanced expression of proinflammatory cytokines, increased microglial activation, especially in the limbic system, has been identified as an important contributor to neuroinflammation in Alzheimer's disease, chronic stress, and depressed suicide victims [20–22]. Recent research supports the role of neuroinflammation in mediating the neurological dysfunction seen with cancer and cancer treatment [11, 14, 15, 23].

The role of immune activation in cancer-related neurological dysfunction becomes especially important when considering cancer therapies which rely in part or in total on activation of the immune system. The efficacy of radiation therapy, especially the abscopal response, is dependent in part on immune activation [24–27]. Direct radiation of the brain has been repeatedly associated with behavioral alterations and impairments in hippocampus-dependent cognition and neurogenesis, effects which may be in part mediated by the immune system [28–32]. However, modeling neurological dysfunction following peripheral radiation in mice has only recently been explored [11].

In addition to radiation treatment, one of the most exciting developments in cancer therapeutics, immunotherapy, also relies on systemic immune activation. Immunotherapeutics have been developed based on findings that show that the immune environment in a tumor prior to treatment is associated with clinical outcomes in many cancers [33, 34]. An enhanced proinflammatory tumor environment is associated with increased survival following conventional cancer therapy, while anti-inflammatory tumor profiles are associated with severely limited survival [33, 34]. Given that radiation therapy has also been implicated as an immune modulator, combined immunotherapy and radiation treatment provides an exciting synergistic possibility to initiate and amplify a beneficial anti-tumor response [16, 24, 35–37].

One immunotherapy demonstrating robust results in combination with radiation treatment involve antibodies against CTLA4 (anti-CTLA4), a checkpoint inhibitor. Anti-CTLA4 induces a proinflammatory environment characterized by increased immune infiltrates in the tumor and combines with radiation therapy to form protective immunity [36, 38]. Pre-treatment with anti-CTLA4 antibodies before radiation therapy is optimal for tumor control in preclinical models [39] and is consistent with successful clinical anecdotes [40, 41] and a retrospective review [42]. Importantly, patients are currently being treated and experiencing sustained remissions with the combination of radiation therapy and anti-CTLA4 immunotherapy [25, 40, 41]. These treatments demonstrate remarkable efficacy with respect to tumor outcomes. However, how these treatments may affect the immune environment in the brain, and behavioral and cognitive outcome measures, is unknown. As yet, very little is understood about the effects of these treatments on the brain, either in healthy individuals or in individuals with tumors, although fatigue has been reported [43, 44].

Clinical studies on cancer-related neurological dysfunction have been limited by the presence of numerous confounders, including genetic variability and related differences in immune function between patients. Mouse tumor models present a unique opportunity to study the consequences of cancer and cancer treatment on brain function in a controlled environment. Establishing a mouse model also allows studying behavioral and cognitive performance alongside tissue and molecular markers. The choice of a subcutaneous tumor model in multiple cell lines allows for precise and reliable comparison of all groups at each stage of tumor development, as compared to a spontaneous tumor model. Placement of the tumor on the hind flank also allows for the best understanding of immunological effects of peripheral radiation while avoiding direct damage to healthy tissues or vasculature that may otherwise confound results due to direct or indirect effects of vascular changes on CNS function [45].

**Figure 1:**
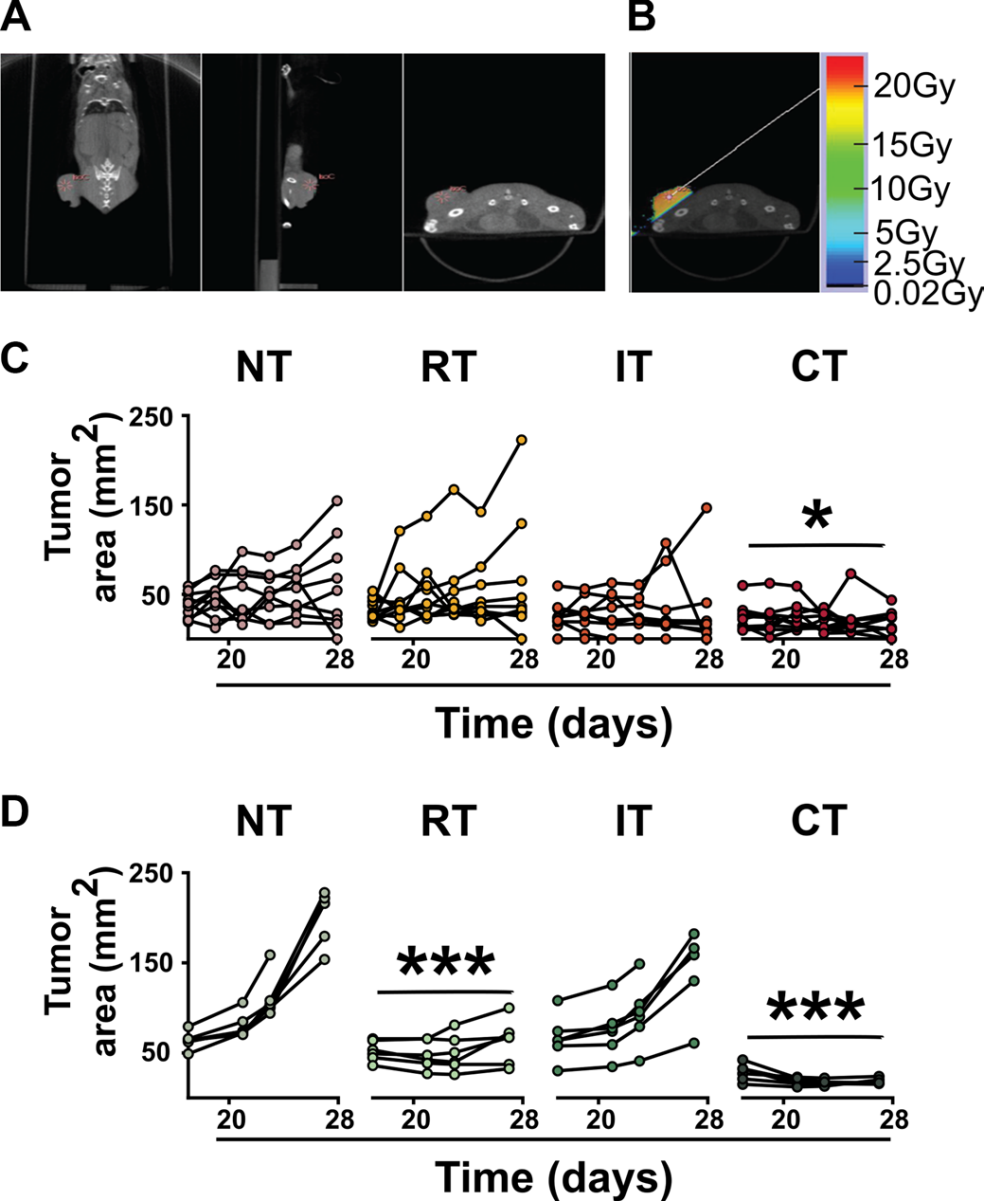
Control of tumor growth achieved with combined treatments (**A**) Tumor visualization and isocenter placement using a cone-beam CT scan with 360 projections on the SARRP. (**B**) Delivery of 20 Gy focal radiation to the tumor site using a 10 × 10 mm collimator at a 50° angle to minimize dose to radiosensitive organs. (**C**) Growth of individual 3LL tumors in C57BL/6J mice on days 15 to 28 in mice left untreated (NT), treated with radiation (RT) or immunotherapy (IT) alone, or combined treatment (CT). Tumor growth in mice receiving CT is significantly slowed compared to those receiving NT. Tumor growth in mice receiving RT or IT is not significantly different than NT. (**D**) Growth of individual CT26 tumors on days 15 to 28 in BABL/c mice in treatment groups described above. Tumor growth in mice receiving RT and CT is significantly decreased compared to NT. *p < 0.05, ***p < 0.001 compared to NT, ANOVA, followed by Dunnett post-hoc (C, *n* = 10 per group; D, *n* = 5).

The current study represents an approach to model cancer-related neurological dysfunction in two important ways. First, although previous models have examined the effects of tumor alone [46, 47], chemotherapy [48, 49], peripheral radiation in healthy mice [11], direct immune stimulation [50, 51], and treatment of cancer-related neurological dysfunction [52, 53], none have examined the effects of peripheral radiation in tumor-bearing mice or of immunotherapy in any tumor state. This is not only pertinent to understand given the emerging potential of these therapeutics, but also for the particular insight they may give to the role of the immune system in general. This study includes both tumor-free and tumor-bearing mice, as well as examines the effects of radiation and immunotherapy both alone and combined (see Figure 1 for the experimental design). Second, this study involves the use of newly developed precision irradiation technologies and the use of distinct genetic backgrounds, which more closely models clinical relevance. The Small Animal Radiation Research Platform (SARRP) combines cone-beam CT-guided imaging with precise radiation delivery and an automatically rotatable stage that allows for delivery of single or multiple beams of radiation to the tumor. This study models cancer-related cognitive dysfunction in two mouse models of cancer: BALB/c mice with colorectal carcinoma and C57BL/6J mice with lung carcinoma. The colon26 adenocarcinoma (CT26) and Lewis Lung carcinoma cell lines were chosen because they are known to secrete IL-6 and TNF-α, do not metastasize when injected subcutaneously, and have growth curves amenable to this experimental timeline [54, 55].

The primary objective of this study was to investigate how checkpoint inhibitor immunotherapy combined with radiation therapy will affect behavioral and cognitive performance in tumor-free and tumor-bearing mice. Here, we show that the combination of radiation and immunotherapy enhances tumor control, but also affects behavioral and cognitive performance, as well as the inflammatory environment in the brain. Immunotherapy and radiation alone also seem to alter the immune environment in the brain, but do not cause the behavioral and cognitive changes seen with combined immunotherapy and radiation. Presence of tumor also affects behavioral and cognitive performance, and the immune environment in the brain. Collectively, these studies show that the neuroinflammatory, behavioral, and cognitive effects of radiation and immunotherapy must be closely considered in the context of cancer-related neurological dysfunction.

## RESULTS

### General health and motor function do not differ between groups

There were no apparent differences in phenotype between the experimental groups, aside from tumor growth in tumor-bearing groups. Initial body weights and body weights taken throughout Experiment 2 (Days 7, 9, 11, 14, 18, and 25) showed no significant differences between groups or within groups across time (Table 1). All groups performed similarly with regard to exploratory activity in the open field and sensorimotor function on the Rotarod (Table 1).

**Table 1:** Body weights, motor function of C57BL/6 with or without 3LL tumors receiving no treatment, radiation alone, immunotherapy alone, or combined radiation and immunotherapy

			Body weight		Motor function	
Tumor status	Treatment	Initial body weight (g) ^a^	Mean body weight (g)^b^	Weight change (g)^c^	Rotarod latency to fall (s)^d^	Open field velocity (cm/s)^e^
Tumor-free	NT	19.36 ± 0.67	19.24 ± 0.54	1.34 ± 1.06	52.61 ± 4.82	5.98 ± 0.19
	RT	20.72 ± 0.44	19.61 ± 0.58	0.84 ± 0.39	52.37 ± 5.15	5.37 ± 0.34
	IT	21.00 ± 0.18	19.96 ± 0.72	1.20 ± 0.39	50.20 ± 4.53	5.47 ± 0.42
	CT	19.58 ± 0.08	18.41 ± 0.82	0.96 ± 0.27	51.61 ± 5.22	5.28 ± 0.35
Tumor-bearing	NT	18.62 ± 0.54	18.85 ± 0.42	1.98 ± 0.66	50.30 ± 4.46	5.50 ± 0.33
	RT	19.86 ± 0.50	19.26 ± 0.51	1.32 ± 0.42	50.37 ± 5.26	5.38 ± 0.26
	IT	19.90 ± 0.49	18.50 ± 1.11	0.76 ± 0.67	53.92 ± 3.84	5.19 ± 0.34
	CT	19.56 ± 0.53	19.28 ± 0.45	0.98 ± 0.62	55.30 ± 4.72	5.82 ± 0.34

Data expressed as mean ± SEM.

^a^Value given is mean initial body weight for animals in Experiment 2. (*n* = 5).

^b^Value given is mean body weight for animals in Experiment 2. (*n* = 4–5).

^c^Weight change was calculated as weight at the end of testing (day 25) minus weight at inoculation (day 0). (*n* = 4–5).

^d^Rotarod latency to fall is the mean time to fall during each of 9 trials. (*n* = 10).

^e^Open Field Velocity is the mean velocity (cm/s) over the course of three 5 minute exposures to the open field arena. (*n* = 10).

### Tumor growth is only controlled in mice receiving radiation and immunotherapy

To evaluate the effects of radiation and/or immunotherapy on growth of 3LL lung carcinoma, tumor measurements (length and width accurately measured with calipers) were taken throughout Experiment 2 (Days 7, 9, 11, 14, 17, 19, 21, 23, 25, and 28) and tumor areas calculated (Figure 1C). Only the combination of anti-CTLA4 checkpoint inhibitor immunotherapy with 20 Gy focal irradiation to the tumor site (referred to as CT) produced a significant decrease in tumor areas as compared to no treatment (NT) (*p* < 0.05, repeated measures). No significant treatment effects were seen in groups treated with radiation (RT) or immunotherapy (IT) alone.

### Decreased measures of anxiety following combined radiation and immunotherapy

**Figure 2:**
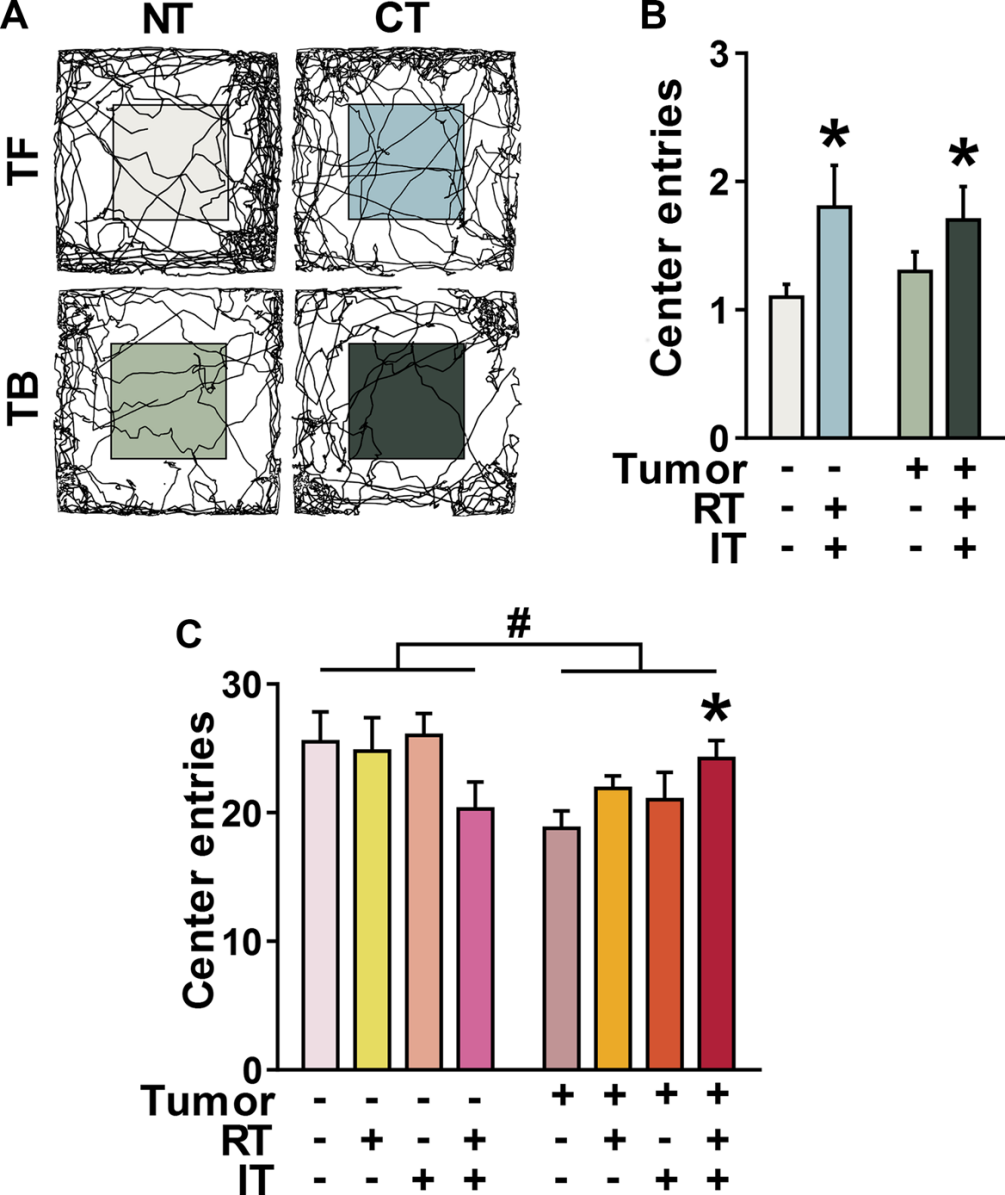
Decreased measures of anxiety mice receiving combined treatment (CT) in two different mouse models of cancer Number of entries into the center of the open field arena was used as a marker for anxiety behavior. (**A**) Representative tracks of open field behavior as recorded in BALB/c mice ± CT26 inoculation left untreated (NT) or treated with CT. (**B**) Both tumor-free (TF) and tumor-bearing (TB) BALB/c mice treated with CT demonstrate decreased measures of anxiety. (**C**) In C57BL/6J mice ± 3LL inoculation, measures of anxiety are increased in TB mice. However, TB mice treated with CT demonstrate decreased measures of anxiety. *p < 0.05 compared to NT, ANOVA, followed by Dunnett post-hoc. #*p* < 0.05, ANOVA, effect of tumor. (B–C, *n* = 10 per group).

Symptoms of anxiety and anxiety disorders are significantly more common in cancer patients than the general population [3, 56]. To assess levels of anxiety following radiation and immunotherapy, the number of entries into the center of the open field was analyzed in BALB/c with colorectal carcinoma (Figure 2A–2B) and C57BL/6J with lung carcinoma (Figure 2C). Representative motion plots of open-field activity in Figure 2A show that BALB/c animals treated with combined radiation and immunotherapy crossed more frequently into the more-anxiety provoking center of the open field. This indicates decreased measures of anxiety in combination treated mice as compared to untreated mice (Figure 2B, p < 0.05, ANOVA). This effect was seen in both tumor-free (TF) and tumor-bearing (TB) mice. However, in C57BL/6J mice, this decrease in measures of anxiety was only seen in tumor bearing mice (Figure 2C, p < 0.05, ANOVA with Dunnett post hoc). Tumor-bearing mice also showed higher measures of anxiety than tumor-free mice (*p* < 0.05, ANOVA). In fact, tumor-bearing mice receiving CT showed restored levels of anxiety on par with those of tumor-free mice receiving no treatment (*p* > 0.05, *t* test). Mice receiving either radiation or immunotherapy alone did not show any significant differences in measures of anxiety when compared to animals receiving no treatment.

**Figure 3:**
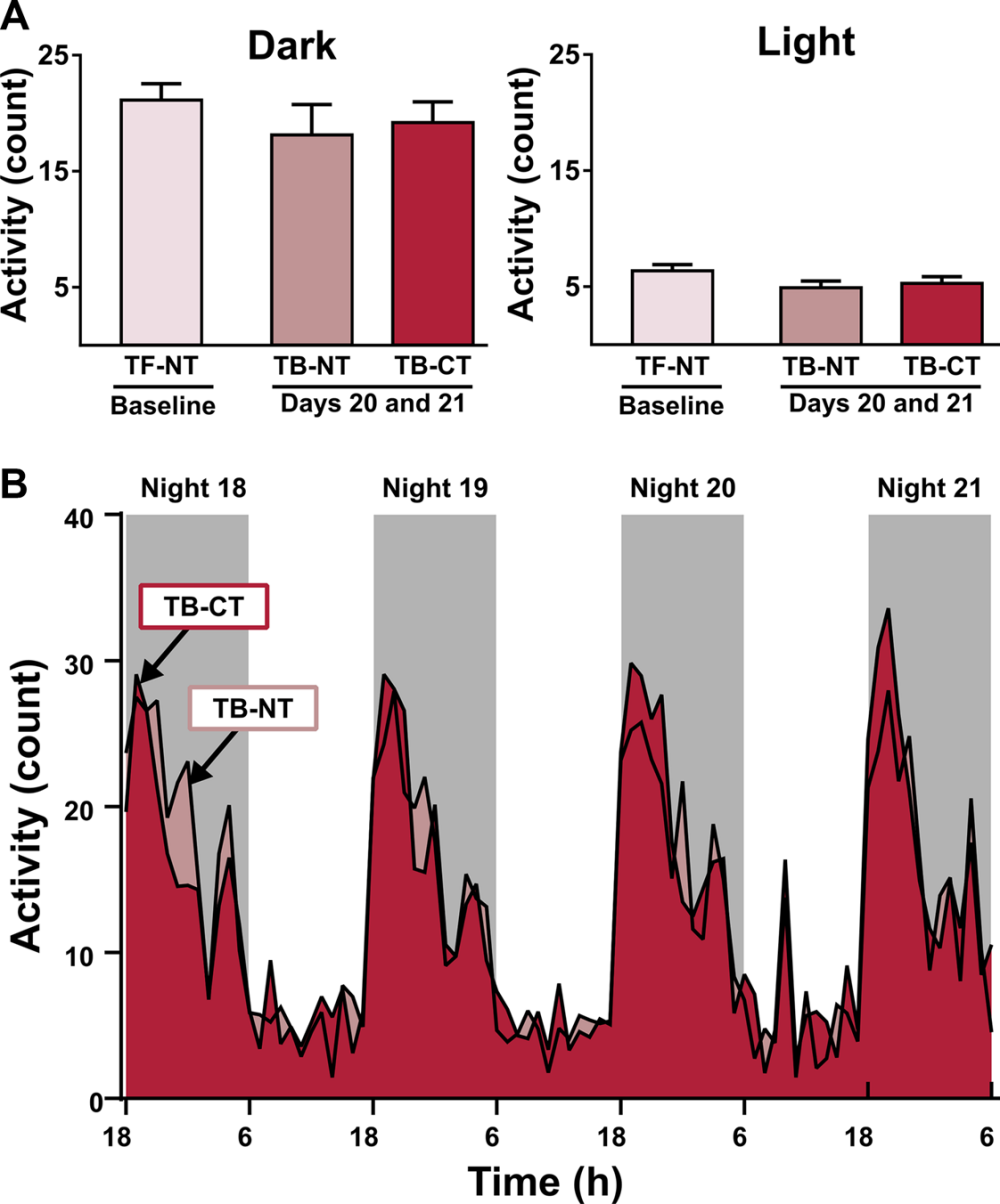
Activity levels do not change with tumor development or treatment with combined immunotherapy and radiation (**A**) Mean activity count was calculated for the light and dark periods of two 48 hr intervals. The first interval was taken over experimental days -1 and -2 and show mean activity levels immediately before tumor inoculation (TF-NT = tumor-free, no treatment). The second interval was taken during the last two days of the experiment, days 20 and 21, and shows mean activity levels in tumor-bearing (TB) mice receiving either no treatment (TB-NT) or combined treatment (TB-CT). There were no significant differences between groups. (**B**) Mean activity patterns showing no significant differences between TB-NT and TB-CT mice during the final 84 hours of Experiment 3.

Other measures of behavior did not show effects of tumor or treatment. Behavioral despair was measured in Experiment 2 by determining the percent time a mouse spent immobile using the forced swim test, during which a mouse is introduced into a water-filled cylinder from which it cannot escape. There were no significant effects of tumor or treatment in percent time immobile during the forced swim test (Table 2). However, tumor-bearing mice demonstrated a significantly longer latency to first immobility (Table 2.) Nest building is a natural rodent behavior that can model disrupted activities of daily living in people with cognitive impairment [57]. Regardless of tumor status or treatment received, mice in Experiment 2 demonstrated similar nest building scores (Table 2). Home-cage activity levels were measured in Experiment 3 through the use of infrared home-cage activity sensors across a 28-day period of four intervals: baseline activity (days –7 to –1), tumor growth (days 0 to 6), post-IT (days 7 to 13), and post-RT (days 14 to 21). There were no significant effects of tumor or treatment on mean activity levels in the light or dark throughout the duration of the experiment (data not shown). Comparing mice at the tumor-free, no treatment (TF-NT) stage with mice after three weeks of tumor growth either receiving no treatment (TB-NT) or combined treatment (TB-CT) also showed no effect of tumor or treatment on home-cage activity (Figure 3A). Mean activity patterns also showed no significant differences (Figure 3B).

**Table 2:** Tumor growth and treatment effects are not seen in all behavioral and cognitive measures

Tumor status	Treatment	Forced swimPercent timeimmobile(%)^a^	Forced swim Latency to firstimmobility (s) ^b^	Nest rating^c^	Fear ConditioningBaseline motion (cm/s)^d^
Tumor-free	NT	70.50 ± 3.52	43.85 ± 6.91	19.24 ± 0.54	265 ± 25.56
	RT	71.67 ± 3.77	34.86 ± 9.31	19.61 ± 0.58	279.9 ± 36.1
	IT	68.84 ± 5.38	26.80 ± 5.51	19.96 ± 0.72	271.6 ± 46.66
	CT	68.89 ± 4.95	37.41 ± 9.39	18.41 ± 0.82	271.4 ± 26.11
Tumor-bearing	NT	72.41 ± 4.20	61.15 ± 10.18	18.85 ± 0.42	266.5 ± 45.12
	RT	58.83 ± 7.15	51.72 ± 10.78	19.26 ± 0.51	269.5 ± 48.08
	IT	65.55 ± 2.75	42.98 ± 8.00	18.50 ± 1.11	267.2 ± 46.96
	CT	69.33 ± 3.80	50.25 ± 6.34	19.28 ± 0.45	243.4 ± 26.17

Data expressed as mean ± SEM.

^a^Percent time immobile based on predominant behavior during 5s time-sampling for 300s. (*n* = 9–10).

^b^Latency to first immobility after placement in forced swim chamber. (*n* = 9–10).

^c^Average nest score from three independent raters. (*n* = 9–10).

^d^Average baseline motion (cm/s) during Fear Conditioning Training session. (*n* = 9–10).

### Combined radiation and immunotherapy induce cognitive impairments in some dimensions in otherwise healthy mice

**Figure 4:**
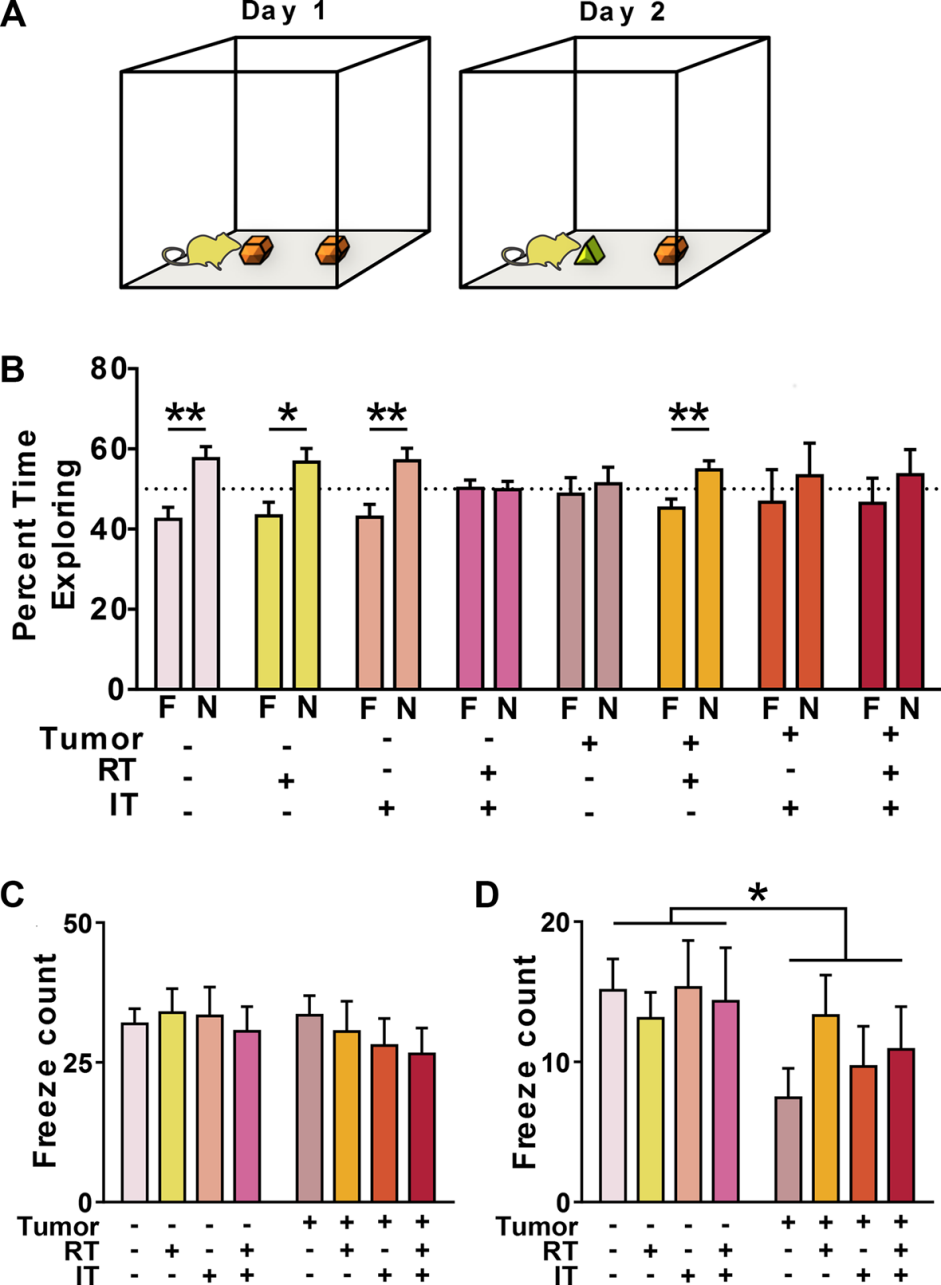
Combined treatment (CT) and presence of peripheral tumor impair memory (**A**) Mice were given access to two identical objects on Day 1. 24 hours later, mice were reintroduced to one familiar object (F) and one novel object (N). Mice recognizing the novelty of the new object spent a greater percentage of their time exploring this object. (**B**) In tumor-free (TF) mice, object recognition was seen in every group except mice receiving combined treatments (CT). In tumor-bearing (TB) mice, object recognition was only restored in mice treated with radiation alone (RT). (**C**) No impairments are seen in a test of contextual fear memory. (**D**) TB mice show impaired cued fear memory as compared to TF mice. *p < 0.05, **p < 0.01, (B, *n* = 10 per group).

Novel object recognition was used to assess cognitive function. Following three acclimation periods, mice were exposed to two identical objects and allowed to freely explore (Figure 4A). Twenty-four hours later, mice were re-introduced to one of the previous familiar objects and a novel object. Except for the combined treatment group, all tumor-free mice showed a robust preference for the novel object (TF-NT, TF-IT *p* < 0.01; TF-RT *p* < 0.05, *t* test). However, tumor-free mice receiving combined radiation and immunotherapy displayed impaired object recognition. Among tumor-bearing mice, only the group receiving RT demonstrated a preference for exploring the novel object (*p* < 0.01, *t* test) (Figure 4B).

Associative memory was assessed by training mice to associate a conditioned stimulus (tone) with a mild foot shock (unconditioned stimulus). Trained mice demonstrate fear of the conditioned stimulus by freezing in place [58]. There were no baseline differences in motion between groups during training (Table 2). To test contextual memory, mice were exposed to an identical environment as that encountered during their training. There were no differences between any of the experimental groups (Figure 4C). To test cued memory, a Pavlovian-type response heavily regulated by the amygdala, mice were placed in a novel environment and exposed to the conditioned tone. Before the tone, all groups exhibited similarly low levels of freezing in response to the novel context (Table 2). However, once the tone was played, mice with tumors demonstrated decreased freezing as compared to tumor-free mice (*p* < 0.05, ANOVA), demonstrating an impairment in cue-dependent fear memory (Figure 4D).

### Immunotherapy elicits enhanced neuroinflammation including activation of microglia, but not neuronal degeneration

**Figure 5:**
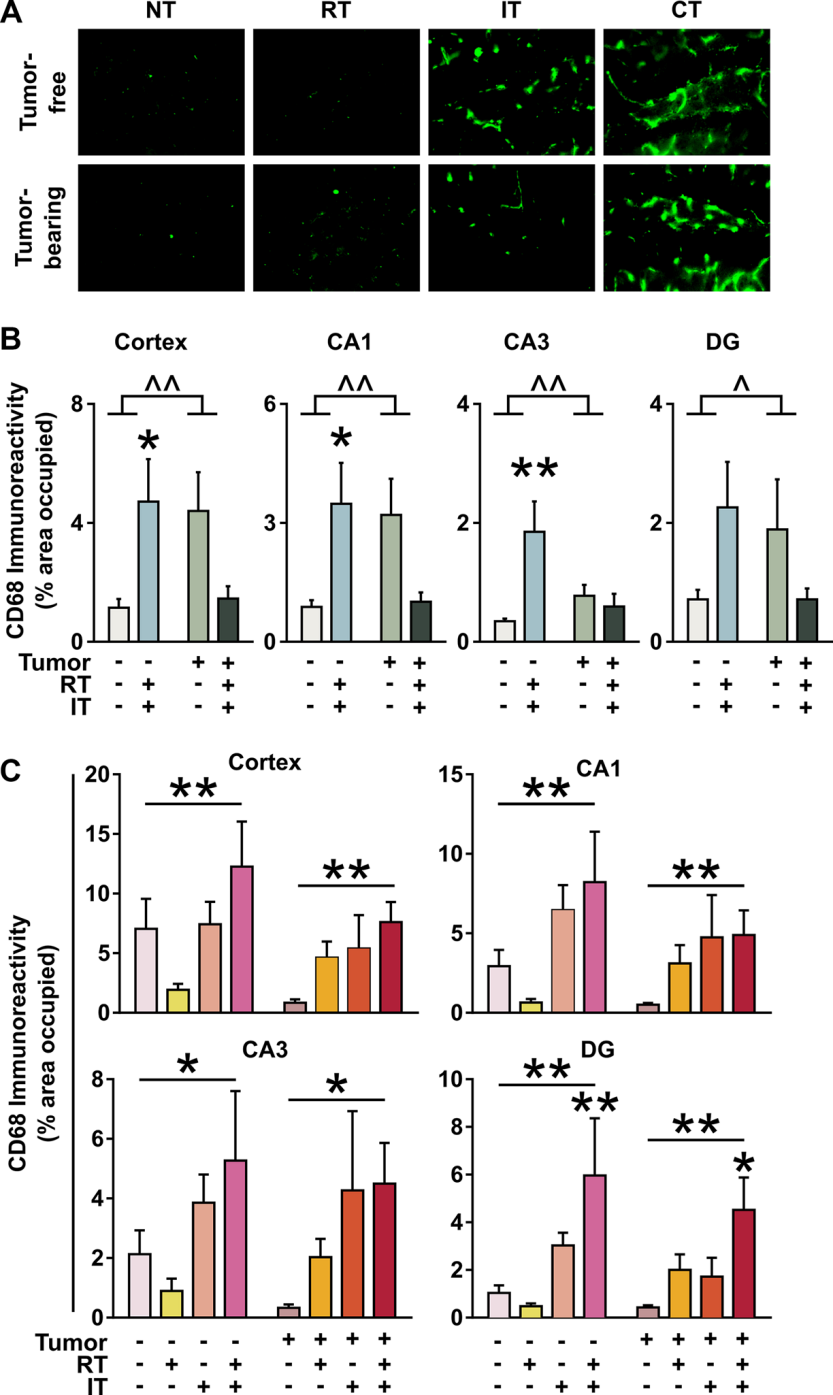
Increased presence of CD68+ activated microglia in the hippocampus and cortex following immunotherapy (IT) or combined treatments (CT) (**A**) Representative images of CD68 immunostaining in the dentate gyrus (DG) of C57BL/6J mice ± 3LL inoculation receiving no treatment (NT), radiation alone (RT), immunotherapy alone (IT), or combined treatments (CT). Quantification of CD68 immunostaining in the cortex and hippocampus (CA1, CA3, DG) of (**B**) BALB/c mice ± CT26 inoculation or (**C**) C57BL/6J mice ± 3LL inoculation receiving NT, RT, IT, or CT. *p < 0.05, **p < 0.01 effect of treatment, ANOVA, followed by Dunnett post hoc. ^*p* < 0.05, ^^*p* < 0.01 interaction, ANOVA, followed by Dunnett post hoc. (B-C, *n* = 6 per group).

Microglial activation has been associated with many neurodegenerative conditions, especially in the limbic system [20, 21]. CD68 was used as a marker of the presence of activated microglia in the cortex and three regions of the hippocampus (CA1, CA3, and dentate gyrus) in the BALB/c mice with colorectal carcinoma (Figure 5A) and C57BL/6J mice with lung carcinoma (Figure 5B–5C). In BALB/c mice, there was increased activation of microglia in the cortex, CA1, and CA3 following combined treatment in tumor-free mice (*p* < 0.05 for cortex and CA1; *p* < 0.01 for CA3; ANOVA with Dunnett post-hoc test). However, a significant interaction between treatment and tumor status was seen when comparing tumor-free to tumor-bearing mice (*p* < 0.05 for dentate gyrus, *p* < 0.01 for cortex, CA1, and CA3; ANOVA). Tumor-bearing mice receiving combined treatment had microglial activation levels similar to that of the tumor-free control (Figure 5A). A similar pattern of microglial activation was seen in tumor-free C57BL/6J mice: there was a significant effect of treatment in the cortex (*p* < 0.01, ANOVA), CA1 (*p* < 0.01, ANOVA), CA3 (*p* < 0.05, ANOVA), and the dentate gyrus (*p* < 0.01, ANOVA). In the dentate gyrus, there was a significantly higher level of activated microglia in mice receiving combined treatment compared to no treatment (*p* < 0.01, ANOVA with Dunnett posthoc). In the C57BL/6J mice, however, a different trend emerged in the tumor-bearing mice than that seen in BALB/c mice. Here, there was a significant effect of treatment (Cortex, CA1, and DG *p* < 0.01; CA3 *p* < 0.05, ANOVA). As seen in the tumor-free C57BL/6J mice, there was a significantly higher level of microglial activation in mice receiving combined treatment compared to mice receiving no treatment in the dentate gyrus (*p* < 0.05, ANOVA with Dunnett posthoc).

**Figure 6:**
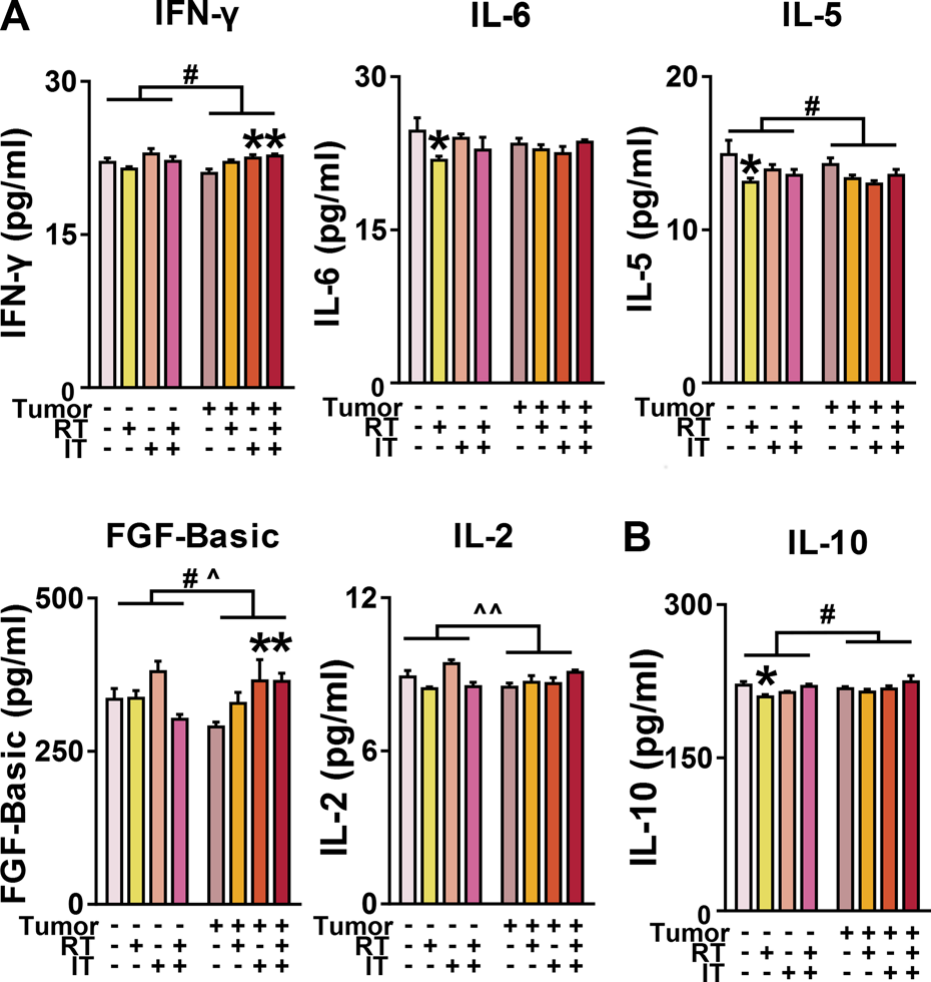
Effect of peripheral tumor, RT, IT, and CT on cytokine, chemokine, and growth factor levels in the cortex and hippocampus (**A**) Changes in cortex expression of IFN-γ, IL-6, IL-5, FGF-Basic, and IL-2. Lewis lung carcinoma inoculation induced changes in IFN-γ, IL-5, and FGF-Basic (*p* < 0.05, ANOVA). There was an effect of interaction of tumor status and treatment on expression levels of FGF-Basic and IL-2 (*p* < 0.05 for FGF-Basic, *p* < 0.01 for IL-2; ANOVA). There was significantly higher expression of IFN-γ and FGF-Basic in tumor-bearing mice receiving immunotherapy (IT) or combined treatment (CT) compared to mice receiving no treatment (NT) (*p* < 0.05, ANOVA with Dunnett posthoc). There was significantly lower expression of IL-6 and IL-5 in tumor-free mice receiving radiation alone (RT) versus NT (*p* < 0.05, ANOVA with Dunnett posthoc). (**B**) Tumor-inoculation increased expression of IL-10 in the hippocampus (*p* < 0.05, ANOVA). Mice receiving RT demonstrate lower levels of IL-10 in the hippocampus (*p* < 0.05, ANOVA with Dunnett posthoc).

Neuroinflammation in the context of cancer and cancer-treatment is frequently characterized by changes in expression of proinflammatory cytokines such as IL- 1β, IFN-γ IL-6, and TNF-α [47, 51]. IFN-γ expression was decreased in tumor-bearing mice in the cortex (*p* < 0.05, ANOVA; Figure 6A). However, IFN-γ levels are increased in the cortex of tumor-bearing mice following immunotherapy alone or combined treatments (*p* < 0.05, ANOVA). Mice treated with RT show lower levels of IL-6 in the cortex as compared to mice receiving NT (*p* < 0.05, ANOVA with Dunnett posthoc). IL-1β and TNF-α levels in the cortex and hippocampus did not vary with tumor status or treatment (data not shown). To further evaluate the neuroinflammatory response seen with peripheral tumor and combined treatment, a number of other important proinflammatory cytokines, chemokines, and growth factors were assessed as well. Of these, IL-5, FGF-Basic, and IL-2 showed significant differences between groups in expression in the cortex. IL-10 was differentially expressed in the hippocampus (Figure 6B).

In order to study the effect of combined therapy on T-cell infiltration of the brain, the occurrence of CD3+ T-cells was assessed via immunohistochemistry. CD3+ T cells were not observed in brain tissue sections of any group.

Fluoro-Jade C is a marker of degenerating neurons. There was no Fluoro-Jade C staining in any group.

## DISCUSSION

Behavioral changes and cognitive impairments commonly accompany cancer and cancer treatment and can have a major impact on patient quality of life [59]. These behavioral changes and cognitive impairments are most likely at least in part mediated by the immune environment in the brain [3, 19, 51, 53, 74]. Because immune activation is at the core of efficacy of critical cancer therapeutics like radiation and immunotherapy, it is essential to understand how these treatments may introduce or exacerbate behavioral changes and cognitive deficits. This is especially important given durable improvements in survivorship achieved by checkpoint inhibitor immunotherapy [44]. We hypothesized that combined treatment with checkpoint inhibitor immunotherapy and peripheral radiation treatment would exacerbate cancer-related neurological dysfunction. In this study, a mouse model of cancer-related neurological dysfunction was used to determine the effects of immunotherapy and radiation treatment on behavioral and cognitive performance, and neuroinflammation in tumor-free and tumor-bearing mice. Here we report that although combined treatment achieves the best tumor control, it is accompanied by behavioral, cognitive, and neuroinflammatory changes.

One novel finding in this study is the change in measures of anxiety elicited by combined treatment. Symptoms of anxiety and depression are significantly more common in cancer patients than in the general population [3, 56]. In our animal models, the effect of peripheral tumor alone on measures of anxiety depended on genetic background and tumor type. C57BL/6J mice with lung carcinoma displayed increased measures of anxiety compared to their healthy controls, reflecting clinical data. However, BALB/c mice with colorectal carcinoma demonstrated no increase in measures of anxiety when compared to healthy controls, a finding which reflects previous studies of behavioral performance in this tumor model [47]. The lack of increase in measures of anxiety in BALB/c mice might be due to their generally lower exploratory activity, making small increases in measures of anxiety as compared to control mice more difficult to detect. Although depression is a common finding in cancer-related neurological dysfunction, our models did not show differences in percent time spent immobile between groups on the forced swim test. Although tumor-bearing mice demonstrated shorter latency to first immobility, this measure is not typically analyzed as the first minute of this test is considered habituation [61, 62]. In future studies, we plan to include more measures of depression-like behavior, including those focused on anhedonia.

Another important novel finding in this study is the cognitive impairment seen with the presence of a peripheral tumor and combined treatment. Deficits in cognitive performance, including impairments in executive function, are frequently reported symptoms in cancer survivors [63, 64]. This cognitive injury has been identified in cancer patients who have not yet been treated [1, 65]. This clinical finding was reflected in our mouse model. We saw impaired performance in both a version of an object recognition test that is hippocampus-dependent and amygdala-dependent cued fear memory in mice with tumors. Of the three treatment arms (radiation alone, immunotherapy alone, and combined treatment), mice with tumors receiving radiation alone demonstrated the greatest recovery of cognitive function following treatment. Interestingly, only combined treatment with radiation and immunotherapy caused impairment of object recognition in otherwise healthy mice. Clinical reports and animal studies indicate that combining radiation and immunotherapy can result in a particularly potent anti-tumor effect [41, 66, 67]. Unfortunately, this potent combination may also have particularly deleterious effects on behavioral and cognitive performance, as shown by the pronounced behavioral changes and cognitive deficits in this group.

There appear to be tumor- or mouse strain- specific differences in the neurocognitive response to treatment. The effect of tumor is shown to vary between strains, but in both cases combined therapy decreased measures of anxiety. Notably, on some measures, there appears to be a differential effect of combination therapy based on tumor status. In the absence of a tumor, combination therapy increased measures of anxiety and microglial activation, depending on genetic background. This observation may have relevance to the very variable individual response to immunotherapy in patient populations as well as the variable emergence of toxicities. BALB/c and C57BL/6 mice are prototypical Th2 and Th1 skewed mouse strains and can have distinct responses to T cell and inflammatory triggers. These results indicate the importance of including healthy mice in assessing the effects of combination therapy on the brain and may have implications for extended use of these therapies beyond the point of achieving clinical cure of disease.

Microglial activation appears consistently increased following treatment with checkpoint inhibitor immunotherapy, although it is not accompanied by neuronal injury. Neuroinflammation is a likely mediator of behavioral changes and cognitive impairments seen with cancer and cancer treatment [46, 64]. Increased microglial activation, especially when chronic, has been associated with neurodegenerative conditions [18]. In both tumor-free and tumor-bearing mice, there was a robust increase in microglial activation depending on the treatment arm. These differences are particularly striking in the hippocampus, which is similar to findings of neuroinflammation in chronic stress and depression [20, 22]. The majority of inflammatory cytokines, chemokines, and growth factors did not show differences between groups. We did, however, observe significant changes based on tumor status in inflammatory cytokines (IFN-γ, IL-6, IL-5, IL-2, IL-10) and a growth factor (FGF-basic). IFN-γ is a critical inflammatory mediator produced by chronically activated macrophages [68]. Cortical levels of IFN-γ were decreased in tumor-bearing mice, but restored to control levels following combined treatment. Decreased expression of IL-5, an important regulator of microglia and infiltrating macrophages in the brain [69], was also identified in tumor-bearing mice and tumor-free mice receiving radiotherapy alone. Associations with IL-5 and cancer-related fatigue have previously been described clinically [70]. Tumor-free mice receiving only radiotherapy also demonstrated decreased cortical expression of IL-6, which has been widely recognized as one of the most important regulators of neuroimmune function and has been repeatedly associated with cancer-related neurological dysfunction [15, 71–73]. Modulation of the hypothalamic-pituitary-adrenal (HPA) axis is a likely mechanism for cognitive and behavioral changes seen with IL-6 expression [74]. Dysregulation of the HPA axis is strongly linked to changes in emotional behaviors [75] and represents an important potential mechanistic explanation for the behavioral changes and cognitive impairments seen in this model. Interestingly, there was a tumor x treatment interaction for the expression of both FGF-basic and IL-2 in the cortex. FGF-basic, which increases with learning and can contribute to recovery from cognitive injury [76, 77], was decreased in tumor-bearing mice, except those receiving immunotherapy, either with or without radiation. IL-2, a hallmark of proinflammatory polarization [78], demonstrated a robust tumor x treatment interaction. IL-2 is a potent inducer of vasopressin release from the hypothalamus [79] and corticotropin-releasing factor (CRF) from the amygdala [80], demonstrating another link to dysregulation of the HPA axis in this model. Finally, IL-10, an important immune inhibitory factor [81], was increased in tumor-bearing mice, but decreased in the hippocampus of mice without tumors treated with radiation only. Taken together, the alterations in expression identified in cytokines and a growth factor point to a significant role of dysregulation of the HPA axis as a potential mechanistic explanation for behavioral and cognitive changes following neuroinflammation in this model.

In this study, central neuroinflammation was identified in association with peripheral radiation and checkpoint inhibitor immunotherapy. In future studies, it would be important to investigate downstream mediators of the effects of neuroinflammation on behavioral and cognitive performance. Previous studies have demonstrated the potential for immune modulation of neurogenesis, neurotransmitter metabolism, and the HPA axis as potential effectors of downstream behavioral and cognitive changes [11, 20]. Future studies are warranted to investigate the causative role of neuroinflammation in mediating behavioral and cognitive effects through the use of anti-inflammatory agents in this model.

In our model, no differences were seen in home cage activity level during the light or dark phases for mice with tumors, regardless of whether they received combined immunotherapy and radiation treatment or not. Although fatigue is another hallmark of cancer-related neurological dysfunction [82, 83], our finding is consistent with previous studies showing that home cage activity is stable in the presence of a peripheral tumor [46, 52]. This also correlates well with the relatively normal performance of mice with tumors and treated mice in tests of general locomotor function and exploratory behavior. These findings indicate that neither tumor growth nor immunotherapy and radiation treatment, under the experimental conditions used, cause dramatic changes in lethargy levels or obvious sickness behavior, as assessed by analyzing home cage activity. Likely, the identified impairments are the result of deficits in higher-level cognition and behavior. We recognize that it is possible that significant fatigue exists, but is not exhibited by changes in levels of home-cage activity. Future studies will utilize voluntary wheel running activity, which has demonstrated differences in fatigue levels in recent models of cancer-related neurological dysfunction [11, 46, 48, 52, 53].

Cancer-related neurological dysfunction is a common and devastating consequence of cancer and cancer-treatment. With improved survivorship and the emerging use of novel therapeutics that may contribute to these effects, prevention and treatment of these behavioral and cognitive impairments is paramount. A better understanding of the role of the immune system in mediating these effects will facilitate the development of treatments to mitigate these effects in the near future.

Developing a model for the behavioral and cognitive changes following immunotherapy and radiation treatment allows us to better understand existing clinical data, expand knowledge of mechanisms, and eventually develop much-needed therapeutics. Much of the existing research on cancer-related neurological dysfunction to date has taken place in breast cancer survivors. Our use of only female mice reflects the preponderance of human data available. Utilizing two genetic backgrounds and tumor types allowed us to examine some of the inter-individual and inter-population differences that are often so significant in translational research projects.

In summary, tumor status has a significant bearing on the behavioral and cognitive effects of treatments. Future efforts are warranted to determine how adjuvant use of immunotherapies may potentiate cancer-related neurological dysfunction.

## MATERIALS AND METHODS

### Study Design

**Figure 7:**
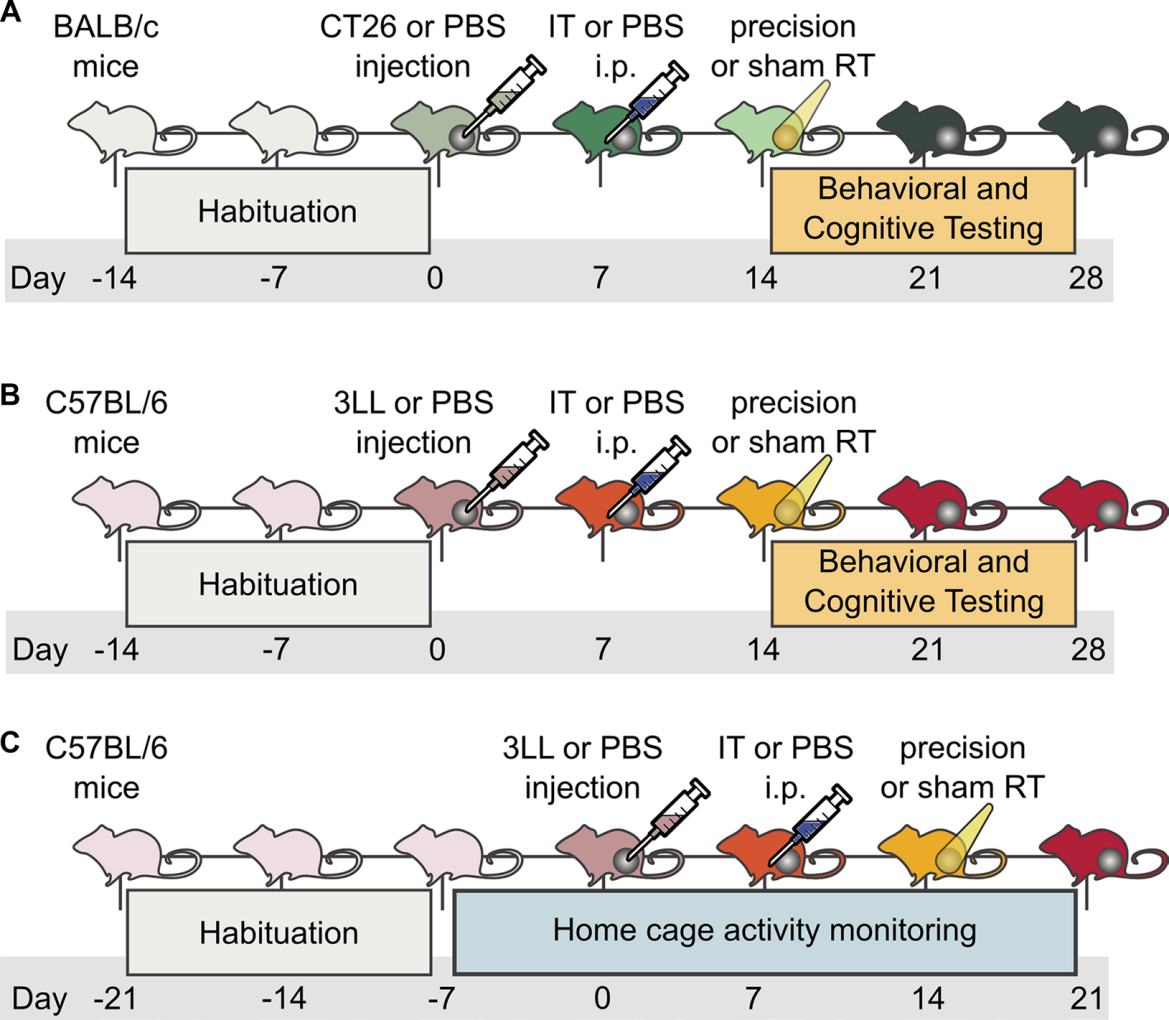
Experimental design (**A**) Experiment 1 involved four groups: 1) tumor-free (TF), no treatment (NT); 2) TF, combined treatment (CT); 3) tumor-bearing (TB), NT; 4) TB, CT. (**B**) Experiment 2 involved eight groups: 1) TF, NT; 2) TF, radiation alone (RT); 3) TF, immunotherapy alone (IT); 4) TF, CT; 5) TB, NT; 6) TB, RT; 7) TB, IT; 8) TB, CT. (**C**) Experiment 3 involved three longitudinal groups: 1) TF, NT; 2) TB, NT; 3) TB, CT; with only groups 2 and 3 present at the end of the experiment.

The study is divided into three experiments (Figure 7). The goal of the *first* experiment was to explore potential neurological effects of combined immunotherapy and peripheral irradiation. The goals of the *second* experiment were twofold: to determine role of 1) immunotherapy and radiation individually and 2) variability introduced by differences in genetic background and tumor type on neurological effects of combination treatment. Experiment *three* was a longitudinal study aiming to examine changes in circadian rhythm and fatigue through tumor inoculation, treatment, and recovery. Mice in Experiments 1 and 2 were transferred from the Earle A. Chiles Research Institute (EACRI) to Oregon Health and Sciences University (OHSU) on experimental day 14. Mice in Experiment 3 remained at EACRI. Throughout the project, logistics and environment were standardized to minimize variation.

### Animals

Female two- to four-month-old wild-type BALB/c (Experiment 1) and C57BL/6J (Experiments 2 and 3) mice were purchased from the Jackson Laboratory (Bar Harbor, ME, USA) and allowed to acclimate to their surroundings for 7-10 d before experiments. The CT26 and 3LL tumors models are derived from female mice and therefore female mice were used by necessity. Female mice are also preferred due to sexual dimorphism in severity of phenotype in tumor-bearing mice: male mice demonstrate greater mortality and weight loss as well as excoriation of the tumor site [47, 84]. Experiments were performed with CT26 colorectal carcinoma which is syngeneic to BALB/c mice and with 3LL lung adenocarcinoma which is syngeneic to C56BL/6 mice. These cancer cells are grown in culture and transplanted to immunocompetent syngeneic mice to generate mouse models of colorectal carcinoma and lung adenocarcinoma. Subcutaneous tumors permit accurate radiation targeting and accurate measurement of response without impeding mouse freedom of motion or function. Rooms were maintained at 21°C on a 12 h light/dark cycle with lights on from 06:00 to 18:00. All testing was performed between 08:00 and 16:00 unless otherwise noted. Normal chow diet (PicoLab Rodent Diet 20, #5053; PMI Nutrition International, St. Louis, MO, USA) and tap water were available *ad libitum*. All procedures were in compliance with the National Institutes of Health Guide for the Care and Use of Laboratory Animals and with IACUC approval at the EACRI and OHSU.

### Cell lines

The CT26 murine colorectal carcinoma [85] (Experiment 1) and the Lewis lung carcinoma (3LL) murine cell line [86] (Experiments 2 and 3) were obtained from the ATCC (Manassas, VA, USA). Stocks were within 3 passages of the ATCC clones with *in vitro* expansion used to generate a bank of matched early passage cells for subsequent experiments. Cells were grown in RPMI-1640 media supplemented with HEPES, non-essential amino acids, sodium pyruvate, glutamine, 10% FBS, penicillin and streptomycin. All cell lines tested negative for mycoplasma by ELISA. To initiate tumor growth, cells were subcutaneously injected into the right flanks on experimental Day 0 at a dosage of 5 × 10^5^ of CT26 (Experiment 1) or 3LL (Experiments 2 and 3) cells suspended in 100 μL PBS per animal. Tumor-free mice were injected with PBS alone using the same procedure. In experiments 1 and 2, half of mice were tumor-free (TF) and half of mice were tumor-bearing (TB). In experiment 3, all mice received tumors.

### Immunotherapy and radiation therapy

On experimental day 7, mice were injected intraperitoneally with either 250 μg Anti-CTLA4 antibody (Clone #9D9, BioXCell, West Lebanon, NH) dissolved in PBS or PBS alone. On experimental day 14, mice received precision peripheral-irradiation or sham-irradiation using the Small Animal Radiation Research Platform (SARRP, XStrahl, Gulmay Medical, Suwanee, GA). Using a cone-beam CT scan with 360 projections, the tumor was visualized and isocenter was placed within the tumor. A single tangent beam using a 5 × 5 mm collimator was utilized to deliver 20 Gy (Figure 2), as previously optimized for the CT26 tumor model [39]. In experiment 1, all mice received either sham treatment (NT) or combined treatments (CT; 4 groups total; *n* = 10 mice/group; 40 mice total). In experiment 2, mice received either NT, peripheral-irradiation only (RT), anti-CTLA4 immunotherapy only (IT), or CT (8 groups total; *n* = 10 mice/group; 80 mice total). In experiment 3, mice received either NT or CT (2 final groups; *n* = 8 mice/group; 16 mice total).

### Behavioral and cognitive testing

Mice in experiments 1 and 2 underwent behavioral and cognitive testing beginning on experimental day 15. Mice were singly housed 24 hours prior to the first behavioral test to minimize the effects of social influences on behavior. As alterations in exploratory behavior and measures of anxiety might affect performance in cognitive tests, they were assessed as well. Behavioral testing was performed in order of increasing stress level [28]. All behavioral testing was performed by an observer blinded to treatment group. The sequence of behavioral testing was: the open field (*n* = 10 mice/group/experiment), Rotarod (*n* = 10 mice/group/experiment), novel object recognition (*n* = 10 mice/group/experiment), fear conditioning (*n* = 10 mice/group/experiment), and forced swim test (*n* = 10 mice/group for Experiment 2).

### Measures of anxiety and exploratory behavior (open field, days 15-17)

The open field consists of a brightly lit enclosure (40.6 × 40.6 cm) with a white plastic floor and clear Plexiglas walls (Kinder Scientific, Poway, CA). On three consecutive mornings, mice were allowed to explore the enclosure for 15 minutes. Movement and exploration were tracked and analyzed with video software from Noldus Information Technologies (Ethovision XT 7, Wageningen, The Netherlands). Distance moved and percent of time spent in the center of the enclosure were analyzed as outcome measures. Number of entries into the center of the enclosure, assessed according to a programmed zone map, was used as a measure of anxiety-like behavior [87]. After each assessment of open-field activity, the equipment was cleaned with 5% acetic acid to remove residual odors.

### Coordination and sensorimotor function (Rotarod, days 15-17)

Mice were placed on an elevated rotating rod (diameter: 3cm, elevation: 45cm; Rotamex-5, Columbus Instruments, Columbus, OH, USA) initially rotating at 5.0 RPM. The rod accelerated 1.0 RPM every 3 s. A line of photobeams beneath the rod recorded the latency to fall (s). For three consecutive afternoons, each mouse received three trials per day with no delay between trials.

### Object novelty recognition (days 18-19)

The object novelty recognition test relies on rodents’ natural curiosity and propensity to orient their attention toward a novel stimulus and is sensitive to hippocampal injury [88]. On day 18, mice were placed into the open field enclosure, identical to as described above, except containing two identical objects (small orange hexagonal prisms) placed 15 cm from the adjacent walls and 10 cm apart (Figure 5). The mice were allowed 10 min to explore objects. On day 19, one of the identical objects (“familiar”) was replaced with a novel object (small green triangular prism) of identical dimensions. Movement and exploration were tracked and analyzed for nose, body, and tail with Ethovision XT 7. Nose point location within the object zone was used to determine exploratory behavior. Object novelty recognition was determined by calculating the percent time spent exploring the novel object out of the time spent exploring the novel and familiar objects.

### Associative learning and memory (days 25-26)

In this task, mice learned to associate a conditioned stimulus (CS, e.g. the environmental context, or a discrete cue-tone) with a mild foot shock (unconditioned stimulus, US). CS-US pairings are preceded by a short habituation period, from which a baseline measure of locomotor activity and other behavior can be scored. Contextual fear conditioning is thought of as hippocampus and amygdala dependent, while fear conditioning to a cue is considered to be amygdala-dependent but hippocampus-independent [89, 90]. Post-exposure freezing, defined as somatomotor immobility with the exception of respiration, is considered a post-exposure fear response, and is a widely used indicator of conditioned fear [58]. Mice were trained and tested using a Med Associates mouse fear conditioning system (PMED-VFC-NIR-M, Med Associates, St. Albans, Vermont) utilizing Med Associates VideoFreeze automated scoring system. Mice were placed inside a brightly lit fear conditioning chamber (100 lux) and allowed to habituate for 90 s. The mice were then exposed to a 30 s (80dB) tone (cue) paired with a 2 s 0.7 mA foot shock administered at 118 s, co-terminating with the tone at 120 s. This series was repeated for a total of five times. Twenty four hours later, contextual associative memory was assessed during re-exposure to the training environment for 300 s. Three hours later, mice were exposed to a modified environment (scented with vanilla extract, novel floor texture covering shock-grid, and a black plastic triangular insert for the walls), allowed to habituate to it for 90 s, and exposed to the sound cue for a period of 180 s. Associative learning was measured as the percent time spent freezing in response to the contextual environment or tone. Motion during shock was measured to account for sensor-motor differences in response to the aversive stimulus.

### Depression-like behavior (day 27)

The forced swim test is commonly used to assess depression-like behavior in animals. It assesses degree of learned helplessness or behavioral despair as determined by the scoring of active (swimming and climbing) versus passive (immobility) behaviors in an inescapable cylinder filled with water [91–93]. In this procedure, mice were placed in cylinders filled with water (24°C to a depth of 15 cm) for 6 minutes and recorded. For the last five minutes of the trial, mice were scored as either immobile or mobile every 5 s. The mice were considered immobile once three paws were immobile and the fourth paw exhibited only minimal movement. Behavioral despair was measured as percent time spent immobile.

### Activity monitoring and nest building

For home cage activity monitoring (Experiment 3), 16 mice were monitored for activity levels using infrared home-cage activity sensors (Biobserve, St. Augustin, Germany). Mice were housed and acclimated to the home-cage environment for three days prior to beginning activity measurement. All 16 mice received 3LL tumor injections one week later. Eight mice were treated with the protocol described above (anti-CTLA4 on day 7, RT on day 14). Activity counts (beam breaks) were measured every second for four weeks and data were expressed as mean activity count per hour. Due to technical problems, data for days 4, 10-13, and 17 were lost. Nest building was measured in Experiments 1 and 2 using an established protocol [94]. Briefly, mice were individually housed in a clean cage and provided two pressed cotton squares (Ancare, Bellmore, NY, USA). Photos of the home cage were taken 48 h later and visually rated by three different blinded scorers on a 5-point nest rating scale.

### Histological evaluation

For histological evaluation, 6 mice from each group from Experiments 1 and 2 were intracardially perfused on experimental day 28 with 20-mL phosphate-buffered saline (PBS) followed by 40mL 4% paraformaldehyde. Brains were removed, stored in 4% paraformaldehyde overnight, and then transferred to 30% sucrose. Fixed brains were coronally sectioned at 40 μm into five series using a cryostat (Microm HM505E, MICROM international GmbH, Walldorf, Germany).

### CD-68, CD-3 immunohistochemistry

CD-68 is a selective marker of activated microglia [95]. Anti-CD3 antibodies can be used effectively as T cell markers as the CD3 protein complex is a defining feature of the T-cell lineage [96]. Coronal brain sections were processed for immunohistochemical detection of CD-68 or CD-3. Sections were rinsed in PBS, incubated in 5% normal goat serum and 0.3% Triton-X in PBS (PBS-TX) for 90 min, rinsed again with PBS, and incubated in primary antisera (CD-68 rat monoclonal: 1:500, Abcam, ab53444, Eugene, OR, USA; CD-3 hamster monoclonal: 1:50, BD Biosciences, 550277, San Jose, CA, USA) in 5% normal goat serum and PBS-TX overnight at 21°C. Sections were then rinsed in PBS and incubated for 2 h in biotinylated goat-anti-rat antibody (for CD-68; 1:200; Life Technologies, Eugene, OR, USA) or goat-anti-hamster antibody (for CD-3; 1:200; Invitrogen, Carlsbad, CA, USA) in PBS-TX. Following rinses in PBS, sections were immediately mounted on slides and coverslipped 3 h later with Vectashield Antifade Mounting Medium with DAPI (Vector Laboratories, Burlingame, CA, USA).

### Fluoro-Jade C staining

Fluoro-Jade C can be used to identify degenerating neurons, regardless of mechanism of injury [97]. Coronal brain sections were prepared for Fluoro-Jade C staining. The sections were mounted on slides and allowed to dry. Slides were immersed in 80% ethanol with 1% NaOH for 5 min followed by a 2 min soak in 70% ethanol and 2 min soak in distilled water. The slides were then incubated in 0.06% potassium permanganate for 15 min, followed by a 2 min soak in distilled water, and 30 min incubation in 0.0001% Fluoro-Jade C working solution. The 0.0001% working solution of Fluoro-Jade C was prepared by adding 10 mL of the stock Fluoro-Jade C solution (0.01%) to 990mL of 0.1% acetic acid in distilled water [97]. The working solution was used within 2 h of preparation. The slides were then rinsed in distilled water and air dried overnight. The air dried slides were then cleared in xylene for 2 min two times before being coverslipped with Permount (VWR, Visalia, CA, USA).

### Cytokine bead assay

For cytokine bead assays, cortical and hippocampal tissues were collected from 4 mice per group from Experiment 2. 25-300 mg of tissue was homogenized in 4.5μL of ice cold PBS containing cOmplete EDTA-free Protease Inhibitor Cocktail (Roche Diagnostics USA, Indianapolis, IN, USA) per mg of tissue. Homogenates were then centrifuged at 13000 g for 15 min and supernatants were transferred and stored at –80°C until used. Cytokine levels in the supernatants were detected using a murine multiplex bead assay (ThermoFisher Scientific, Waltham, MA, USA) and read on a Luminex 100 array reader. Cytokine concentrations for replicates of each tissue sample were calculated according to a standard curve. Tested proinflammatory markers included cytokines (GM-CSF, IFN-γ, IL-1α, IL-1β, IL-2, IL-4, IL-5, IL-6, IL-10, IL-12 (p40/p70), IL-13, IL-17, TNF-α), chemokines (IP-10, KC, MCP-1, MIG, MIP-1α), and growth factors (FGF-basic, VEGF).

### Statistical analyses

Data are expressed as mean ± standard error. Multiple groups and/or multiple time points were analyzed using two-way ANOVAs followed by Dunnett or Tukey-Kramer *post hoc* tests when appropriate using Graph Pad Prism software (San Diego, CA, USA), or repeated-measures ANOVA using SPSS software (Chicago, IL, USA). Only two-tailed *t*-tests were used. In addition to effects of tumor and treatment, interactions between effects of tumor and treatment were also assessed. A probability value of less than 0.05 was considered significant.
